# Outcome of proximal femur shaft fractures in school going children treated with locking compression plates

**DOI:** 10.12669/pjms.37.5.3938

**Published:** 2021

**Authors:** Faaiz Ali Shah, Mian Amjad Ali, Naeemullah

**Affiliations:** 5 Dr. Faaiz Ali Shah, FCPS. Department of Orthopaedics & Traumatology, Lady Reading Hospital, Peshawar, Pakistan; 6 Dr. Mian Amjad Ali, PhD. Department of Orthopaedics & Traumatology, Lady Reading Hospital, Peshawar, Pakistan; 7 Dr. Naeemullah, FCPS. Department of Orthopaedics & Traumatology, Lady Reading Hospital, Peshawar, Pakistan

**Keywords:** Locking compression plate, Proximal femur shaft fracture, Subtrochanteric fracture

## Abstract

**Objectives:**

To determine the clinical and radiological outcome of proximal femur shaft fractures in school going children treated with locking compression plates (LCP).

**Methods:**

This descriptive study was conducted in Orthopaedic Division Lady Ready Reading Hospital Peshawar from 25^th^ June 2018 to 25^th^ September 2020. Children of either gender and age 6 to 12 years old with subtrochanteric and proximal one third femur factures fulfilling the inclusion criteria were enrolled in this study. Open reduction and internal fixation with 4.5 mm narrow locking compression plates (LCP) were done in all. Post operative clinical outcome was evaluated by using Flynn scoring system and graded as excellent, satisfactory and poo results. Radiological assessment of fracture union was done through anteroposterior (AP) and lateral X-ray radiographs.

**Results:**

A total of 60 children with mean age 9.01±1.61 SD (range 6 to 12 years) were included in our study. Oblique fractures were present in 23(38.3%) children, spiral in 20(33.3%), transverse in 11(18.3%) and comminuted in 6 (10%) children. The radiological union time was 13.3±1.2 weeks (range 9.4 to 18 weeks). Majority (88.3%, n=53) of children had excellent clinical outcome according to Flynn’s scoring system while satisfactory outcome was noted in 7(11.6%) children. No cases of delayed union, mal union, nonunion and implant failure was reported.

**Conclusion:**

The results of our study indicated that proximal femoral shaft fractures in school going children treated with locking compression plates had excellent clinical and radiological outcome. We therefore recommend locking compression plate as the implant of choice to fix proximal femoral shaft fractures in school going children.

## INTRODUCTION

Subtrochanteric femur fractures in children accounts for approximately 4 to 10% of all femur fractures.^1^ These fractures are caused by high energy trauma like road traffic accidents and fall from significant height.^2^ Since there is no consensus on the exact definition of subtrochanteric fractures, many authors combined proximal femoral shaft fractures with subtrochanteric fractures for management purpose.^3^ Flexible intramedullary nailing is the most commonly used technique to treat femoral shaft fractures in children.^4^ However, length wise unstable subtrochanteric and proximal fractures particularly in older and obese children when treated with elastic intramedullary nails results in high complication rates like delayed union, malunion, limb length discrepancy and refracture after implant removal.^5^

These fractures are treated with locking compression plates(LCP) to avoid excessive per operative radiation hazards and to minimize the post operative complications of flexible intramedullary nails.^2^ It is a stable fixation with strong torsional and axial stability and has very low frequency of complications.^6^,^7^ Locking compression plates are preferred over conventional stainless steel dynamic compression plates (DCP) to treat these fractures because they are usually close to the proximal metaphysis and physis thus allowing a very limited space for internal fixation and with very low screw purchase.^8^,^9^ Another advantage of locking compression plate in this region is the freedom of using epicortical screws which avoids injury to the physis and penetration into the joint space, minimizing surgical time, stress riser and neurovascular injuries.^10^ Locking compression plating is a safe and effective alternative to elastic intramedullary nailing for proximal femoral shaft fractures in school going children with excellent post operative clinical and radiological outcome.^11^

The objective of our study was to determine the clinical and radiological outcome of proximal femur shaft fractures in school going children treated with locking compression plates. The results of our study will help us in formulating guidelines for treating subtrochanteric and proximal femur shaft fractures in school going children in our set up.

## METHODS

This descriptive study was conducted in Orthopaedic Division Lady Ready Reading Hospital Peshawar, from 25^th^ June 2018 to 25^th^ September 2020. The study protocols were approved by the Ethical Review Board of Lady Reading Hospital (Ref No.: 347/LR, dated May 4, 2018). Children of either gender and age 6 to 12 years of age with closed subtrochanteric femur fractures (Fielding Type-II and Type-III fractures)^12^ and proximal one third femoral shaft fracture presented within a week were enrolled in our study. Radiologically fractures were classified^2^ as spiral, oblique, transverse and comminuted (Winquist Grade 0, I, II).^13^ Pathological fractures, bilateral femoral fractures, floating knee injuries and polytrauma children requiring surgical intervention for head, chest and abdomen were excluded. Informed written consent was taken from parents of children.

Open reduction and internal fixation with locking compression plates (LCP) were performed under general anaesthesia. Preoperative second-generation intravenous cephalosporin (Cefuroxime) was given. The fracture was reduced under vision and lagged (for spiral and oblique fractures). An appropriate length 4.5mm narrow straight titanum locking compression plate (LCP) was utilized to stabilize the fracture with at least six cortices above and six below the fracture. Unicortical locking screws were inserted for fractures near the physis or joint. All the surgeries were done by the same team of expert orthopaedic consultants. Check X-ray was advised next day. Post operatively hip, knee and ankle exercises were started by physiotherapist on first post operative day and patient was discharged. Intravenous antibiotics were continued for three days.

Post operatively all the patients were assessed by an orthopaedic consultant other than the operating surgeon in outpatient department every 2^nd^ week for initial three months and then every 3^rd^ month until one year. In each visit clinical outcome was evaluated by using Flynn scoring system^14^ (Table-I) and graded as excellent, satisfactory and poo results. Radiological assessment of fracture union was done through anteroposterior (AP) and lateral X-ray radiographs. The fracture was labelled as united when radiologically callus formation was noted on at least three out of four cortices on AP and lateral X-rays with no visible fracture line and clinically no tenderness and pain on palpation of the fracture site and on weight bearing. The fracture had delayed union when no signs of callus was seen at three months post operatively and pain and tenderness persisted. Fractures were termed non unions if no radiological callus was observed at 6^th^ months after surgery.^2^ Locking compression plates were removed 12^th^ week after complete radiological union provided the child has full range of hip and knee motion, painless weight bearing and squatting.

**Table-I:** Flynn outcome scoring system.

S. No	Parameter	Excellent Result	Satisfactory Result	Poor Result
1	Malalignment	< 5 Degrees	6 to 10 Degrees	More tha10 degrees
2	Limb-Length Discrepancy(LLD)	< 1 Cm	1 to 2 Cm	More than 2 Cm
3	Pain	None	None	Present
4	Complications	None	Minor or resolved	Major Complication/Morbidity

The data was analyzed with SPSS version 20. Important quantitative variables like age and union time was presented as mean and standard deviation while qualitative variables like gender, cause of fracture and side of fracture was presented as frequencies and percentages. The data was presented in table and graph where necessary.

## RESULTS

The total number of patients in our study were 60. The mean age was 9.01±1.61SD (range 6 to 12 years). Male were 43(71.6%) and female 17(28.3%). Right femur was fractured in 39(65%) and left in 21(35%). Majority (71.6%, n=43) of fractures were caused by motor vehicle accidents while 17(28.3%) children sustained fractures due to fall from height. Fielding Type-II fracture was noted in 9(15%), Type-II in 22(36.6%) and proximal one third femur in 29(48.3%). The patterns of fractures were oblique in 23(38.3%) spiral in 20(33.3%) transverse in 11(18.3%) and comminuted in 6 (10%). Most (51.6%, n=31) children were received in hospital within 24 hours of sustaining the fracture, 22(36.6%) in 24 to 48 hours and 7(11.6%) in 48 to 72 hours. The average radiological complete union time was 13.3±1.2 weeks (range 9.4 to 18 weeks). Partial weight bearing was allowed at 6^th^ week post operatively while full weight bearing at 12^th^ weeks and onwards. Majority 53 (88.4%) of patients had excellent clinical results according to the Flynn scoring system while satisfactory results were noted in 7(11.6%) patients. No poor results were reported. Hip and knee range of motion were normal. All fractures united. No cases of delayed union, mal union, nonunion and implant failure was reported. Superficial skin infection was reported in 4(6.6%) patients which was resolved with daily dressing and antibiotics. Limb length discrepancy (LLD) was noted in 3(5%) children. Shortening of about 1.5 cm was noted in 1(1.6%)and lengthening of about 1.5 cm in 2(3.3%) children. The average hospital stay was 6±2 days (range two to 10 days). Plates were removed in 41(68.3%) patients (average 25.3th week post operatively, range 20.4 to 40.2 weeks). The removal was easy in 23(56%) patients and difficult in 18(43.9%). The difficult plate removal patients were cases with post op duration of 32 weeks. No refracture was reported on average follow up of 12^th^ weeks after plate removal.

**Figure:**
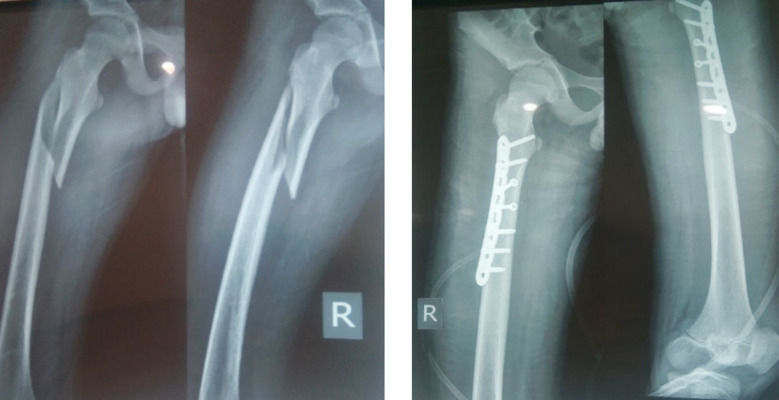
Radiographs-I & II: Pre operative and post operative radiographs of a 9 year old boy.

**Figure:**
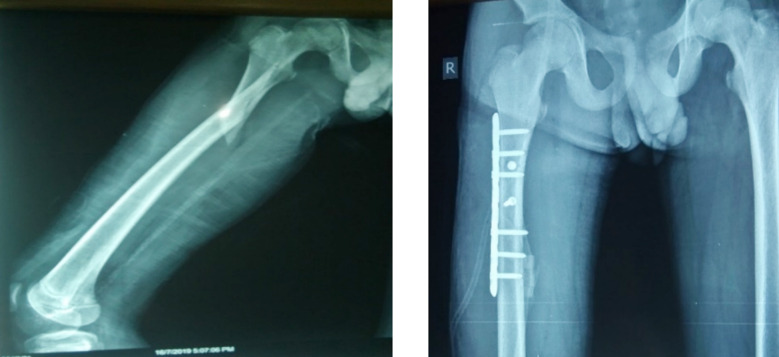
Radiographs-III & IV: Pre operative and post operative radiographs of 11 year old boy.

## DISCUSSION

Good outcome results have been reported in paediatric subtrochanteric and proximal femur shaft fractures treated with open reduction and internal fixation with locking compression plates (LCP).^15^ Studies have shown that LCP is biomechanically more stable construct than elastic intramedullary nailing for treating paediatric proximal femoral shaft fractures in school going children.^16^ In our study excellent post operative results have been reported in 88.3% and satisfactory in 11.6% patients. Our results are comparable with other international studies. Li Y et al^11^ documented excellent results in 87%, satisfactory in 10% and poor in 3%. Xu X et al^17^ noted excellent outcome in 87% children while Wu QZ et al^18^ reported excellent results in 76.9%, good in 15.3% and fair in 7.6% children. Some authors^19^ are of the opinion that although plating is a rigid form of fixation it has minimal complications and produced excellent results in 67.8% and satisfactory in 32.1% children. In our study all the children achieved complete radiological union in 13.3±1.2 weeks (range 9.4 to 18 weeks). Variable union times have been reported in previous studies. Luo^2^ noted union in 2.63±0.77 months, Li Y et al^11^ in 11.4 weeks, and Jolly A et al^20^ in 10.7 weeks. We could not found any correlation between the type of fracture or grades of comminution to the time of union, implant removal or complication. The reasons could be the less number of overall comminuted fractures (10%, n=6) in our study and the plates were not removed in all patients but only in 41(68.3%) patients and out of which only one fracture was comminuted. Luo Y et al^2^ however had reported that comminuted fractures were earlier to unite (p< 0.001) and had earlier plate removal(p=0.006) than other types of fracture.

We had observed superficial skin infection in 4(6.6%) children and limb length discrepancy (LLD) in 3(5%) children. Shortening of about one cm was noted in 1(1.6%) and lengthening of about 1.5 cm in 2(3.3%) children. Studies have shown that although small overgrowth of femur fracture is common in children, limb length discrepancy of more than two cm can affect child’s normal posture and walking and require surgery for correction.^2^ May C et al^21^ and colleague did a detailed analysis of post-operative complications of paediatric femoral fractures fixed with plating. They classified the post-operative complication into major and minor complications. Major complications required unplanned surgery while minor complications resolved with conservative treatment. They reported 6% major complications (1 limb length discrepancy, 2 deep infections and 2 valgus deformities) and 7% minor complications (1 superficial skin infextion, 2 cases of screw prominence with pain and one minor asymptomatic malunion in valgus position), They were of the opinion that since major complications developed later than minor complications (29.1 months versus 12.5 months) long term follow up must be ensured to detect and treat these complication promptly.

In our study we adopted the open method of plating to fix subtrochanteric and proximal one third fractures (Radiographs I-IV). Some studies^11^ compared open method with submuscular plating but found no statistical difference in outcome score (93% versus 100%). However, increased amount of blood loss with open plating and asymptomatic rotational deformities with submuscular plating had been reported.^6^ May C et al^21^ are of the opinion that often intra operative conversion of sumuscular plating to open plating is required when the surgeon is unable to reduce the fracture accurately with submuscular approach.

Two possible issues of LCP application needs to be mentioned here. First, the cost of titanum locking compression plate can be an important issue particularly in developing countries like Pakistan where LCP cost is four times that of conventional plates.^22^,^23^ Many authors therefore advocated that LCP application should be reserved in cases where conventional stainless compression plates cannot provide optimum fixation.^22^ The second issue is whether LCP should be removed routinely or not? Various indications for LCP removal in children include concern of the parents or surgeon, hardware prominence, children with significant growth remaining and plates located within 2cm of the physis.^21^,^24^ We had removed LCP in 41(68.3%) patients (average 25.3th week post operatively, range 20.4 to 40.2 weeks). The removal was easy in 23(56%) patients and difficult in 18(43.9%). Difficulty in removal could be due to screws cold welding to plates, excessive bone ingrowth or distortion of the screw head.^10^ In our opinion this complication can be avoided by relatively early removal of LCP after fracture union. However, further studies need to be done to determine the safe duration of LCP removal after fracture union.

We had not documented any refracture after plate removal on an average follow up of 12^th^ weeks after plate removal. Becker T et al^24^ reported 3(8.1%) refractures within average of 18 days after LCP removal. He concluded that LCP is as stiff as external fixator and risk factors for refracture needs further evaluation.

### Limitations of the study

First, the design of our study was descriptive rather than comparative. Second, we could not analyze some variables of our study like surgery time, blood loss and body mass index. Third, our follow up after plate removal was short. We recommend further studies to address all such limitation and verify our results.

## CONCLUSION

The results of our study indicated that proximal femoral shaft fractures in school going children treated with locking compression plates had excellent clinical and radiological outcome. It had minimum complications. No image intensifier was needed in these fixations. We therefore recommend locking compression plate as the implant of choice to fix proximal femoral shaft fractures in school going children.

### Authors` Contribution

**FASH** conceived, designed and did statistical analysis & editing of manuscript.

**MAA** did review and final approval of manuscript, he is also the accountable for the accuracy or integrity of the work.

**NU** did data collection and manuscript writing.
